# Supplementary figures and images for: Sir2 and Reb1 antagonistically regulate nucleosome occupancy in subtelomeric X-elements and repress TERRAs by distinct mechanisms
Source: PLoS Genet. 2022 Sep 22;18(9):e1010419. doi: 10.1371/journal.pgen.1010419 (PMC9531808; doi:10.1371/journal.pgen.1010419)

## TEL1L

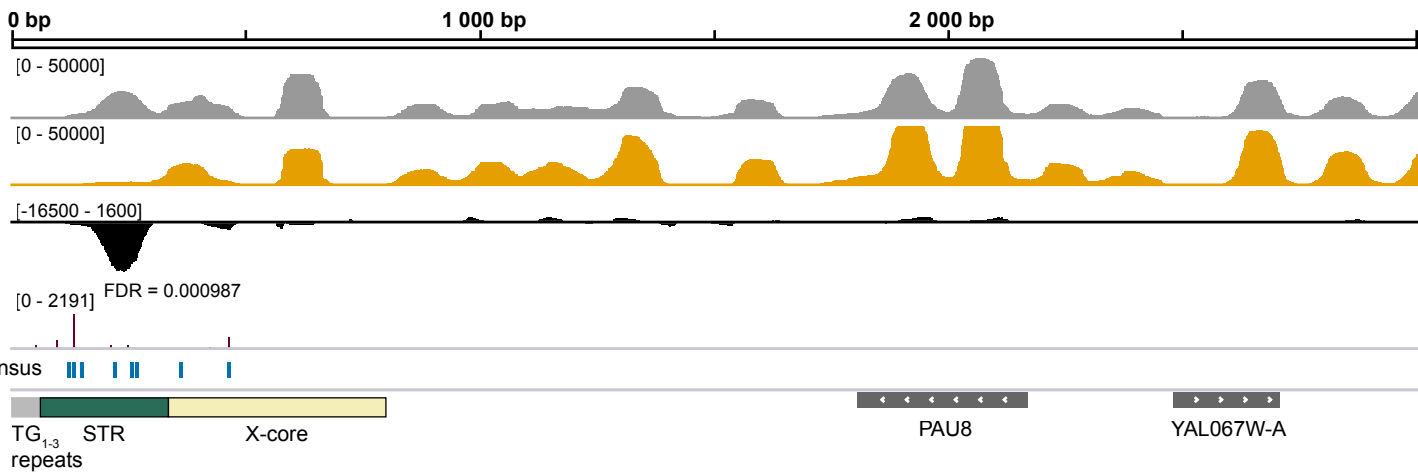

## TEL2L

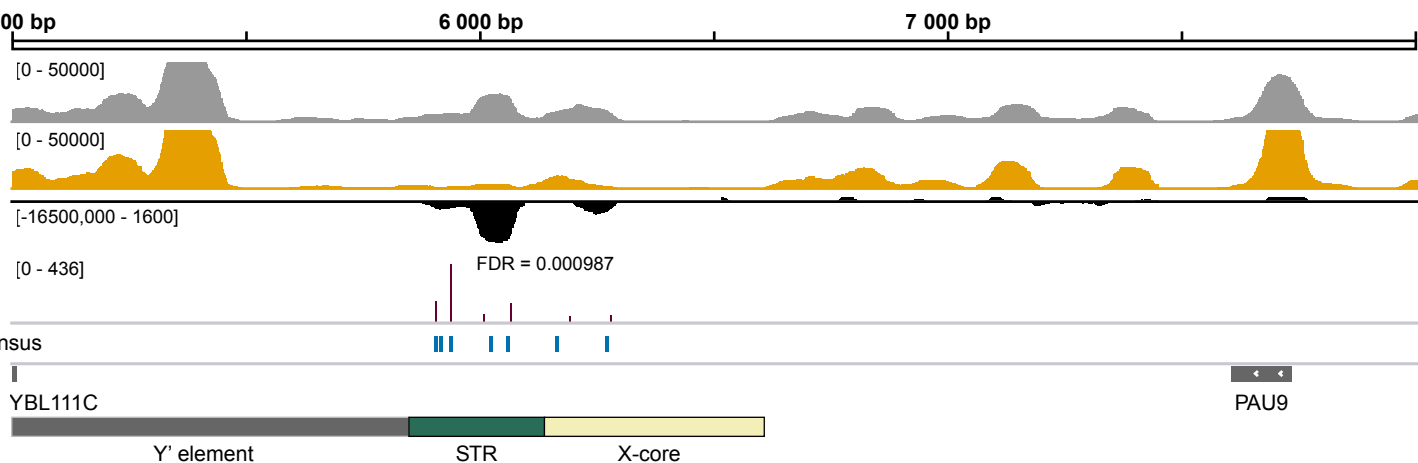

## TEL2R

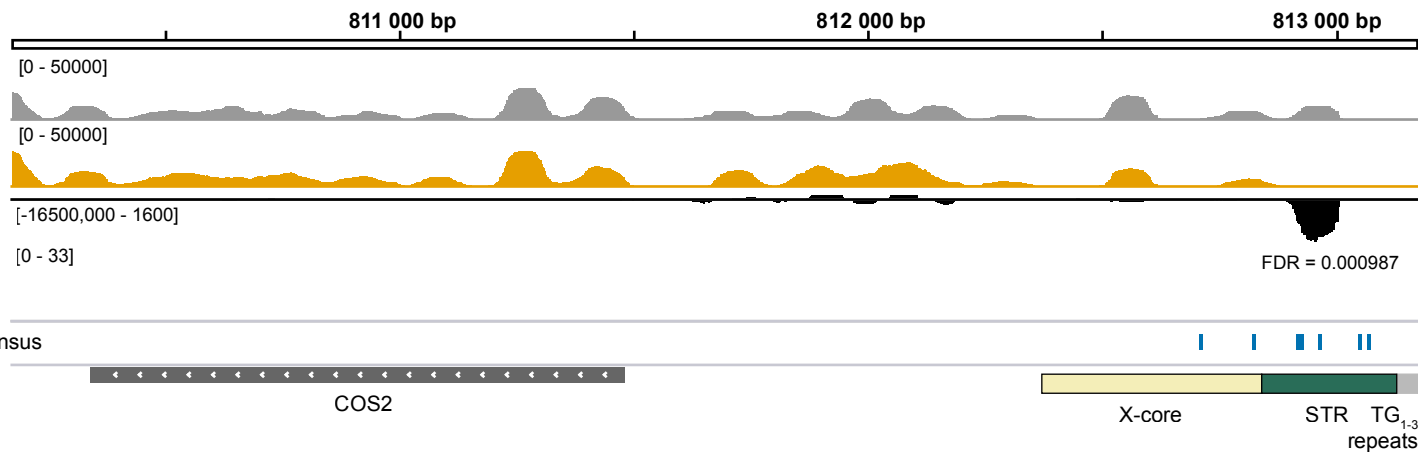

## TEL5L

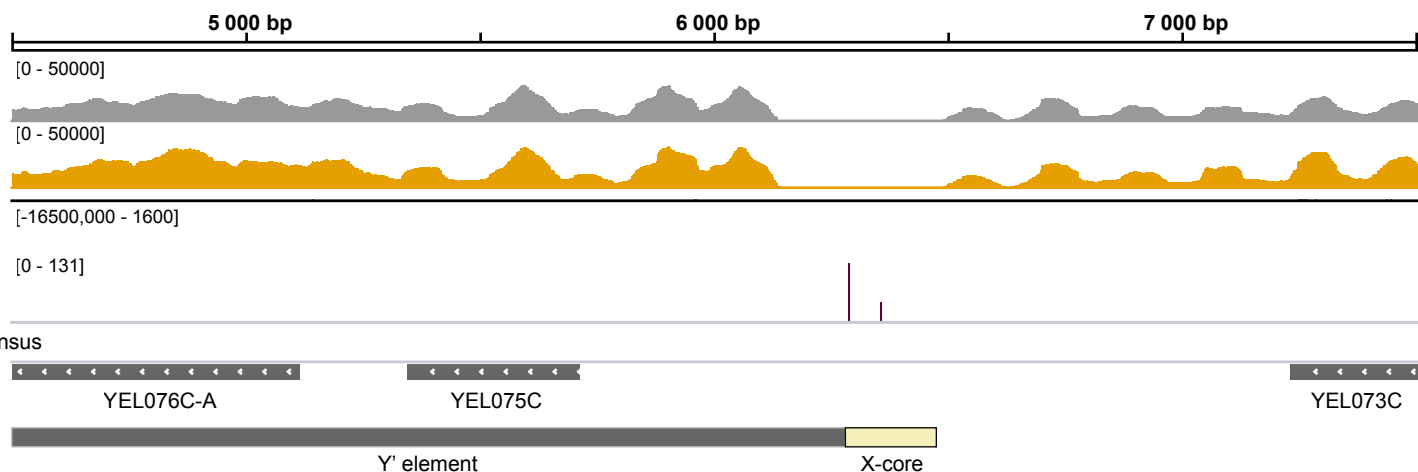

Supplement: S1 Fig — Illustrations of a 3000 bp region of the indicated telomeres in the Integrative Genome Viewer (IGV). Shown are tracks generated from MNase-seq tracks of WT (grey), sir2 (orange) and the DANPOS-calculated difference between sir2 and WT (black), including FDR values when applicable. Reb1 consensus sites are denoted in blue and Reb1 occupancy levels (ChIP-exo) are shown in dark red. Genes are shown in the bottom track in grey. (PDF) [file pgen.1010419.s005.pdf]

**TEL5R**

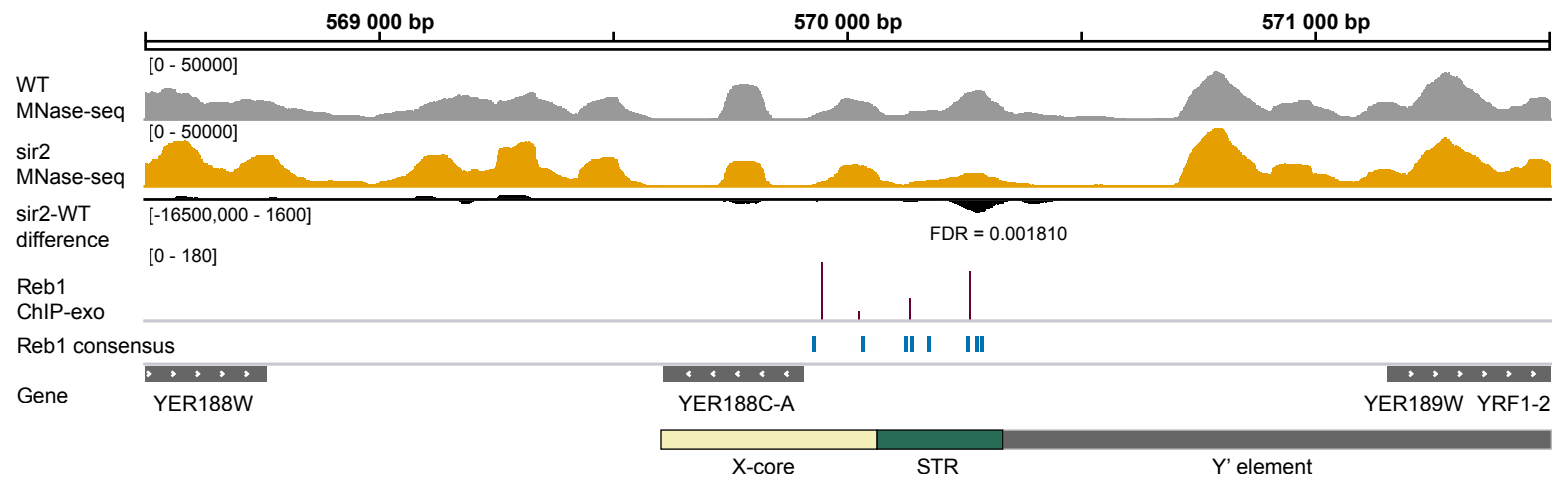

**TEL6L**

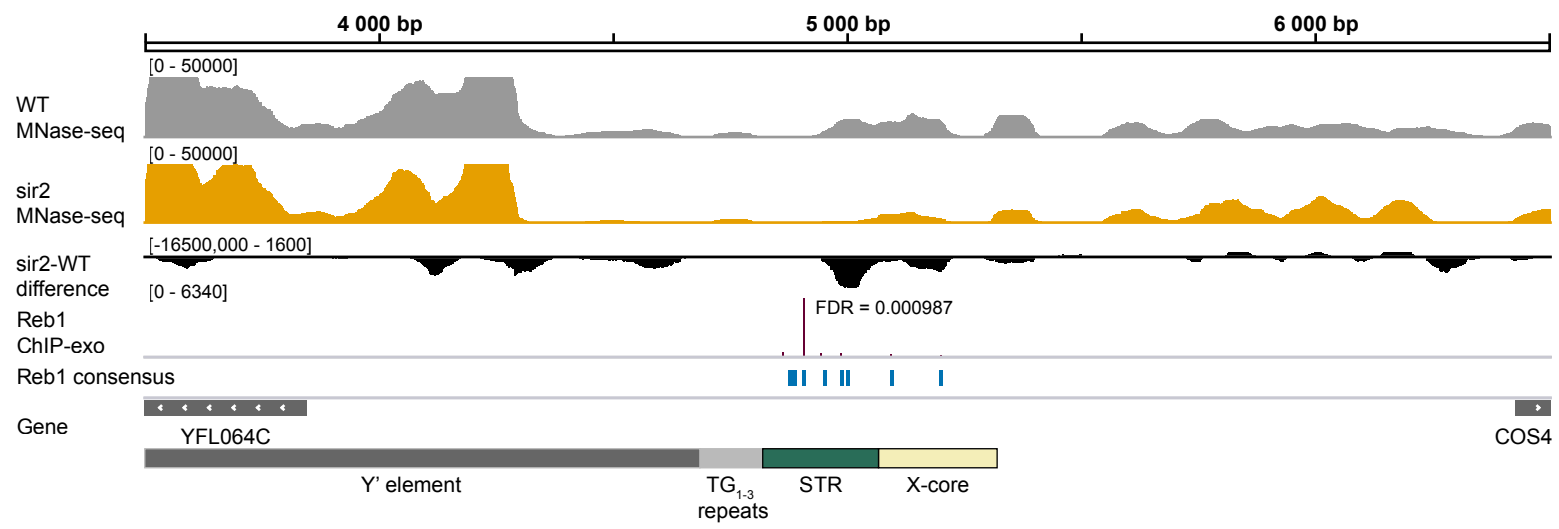

**TEL6R**

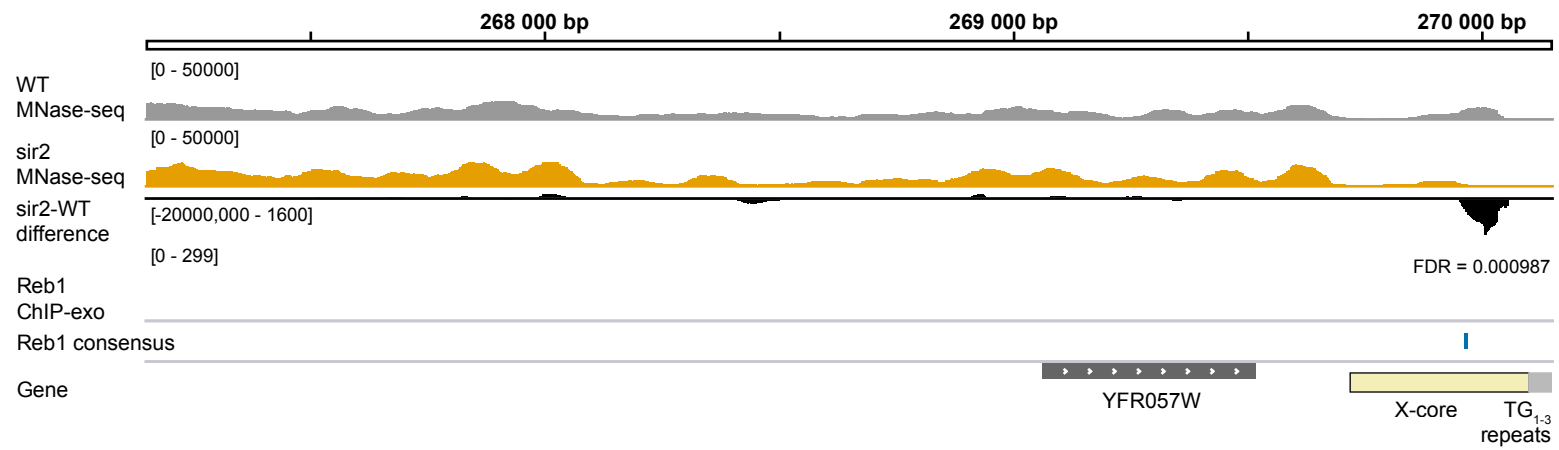

**TEL7R**

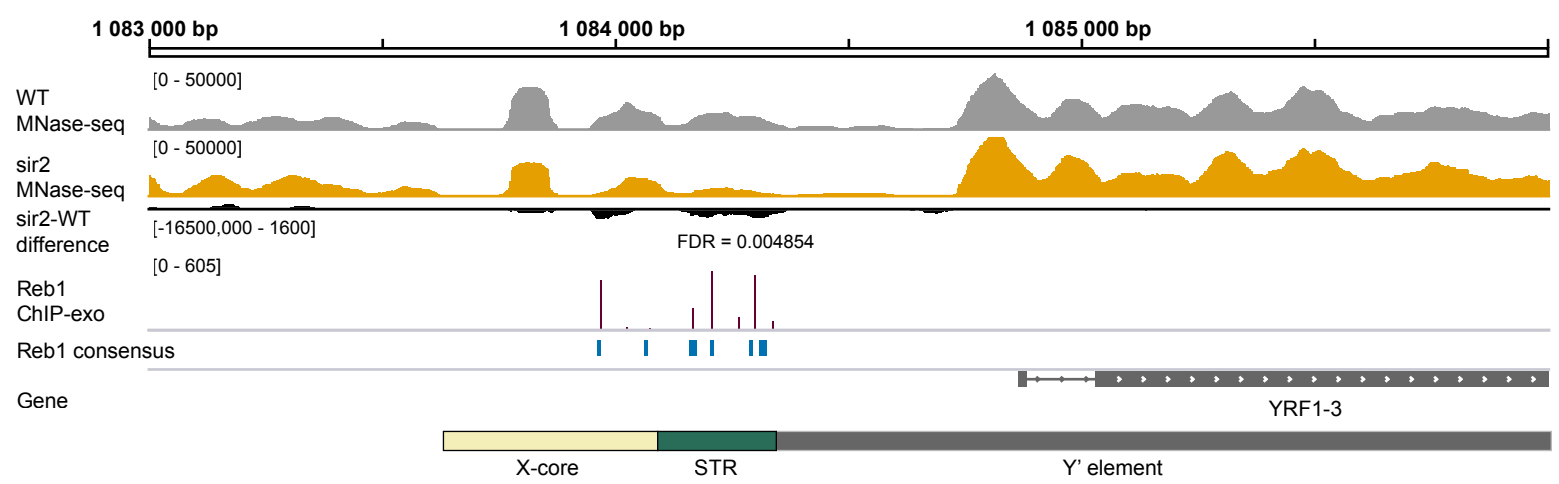

Supplement: S2 Fig — (PDF) [file pgen.1010419.s006.pdf]

## TEL8L

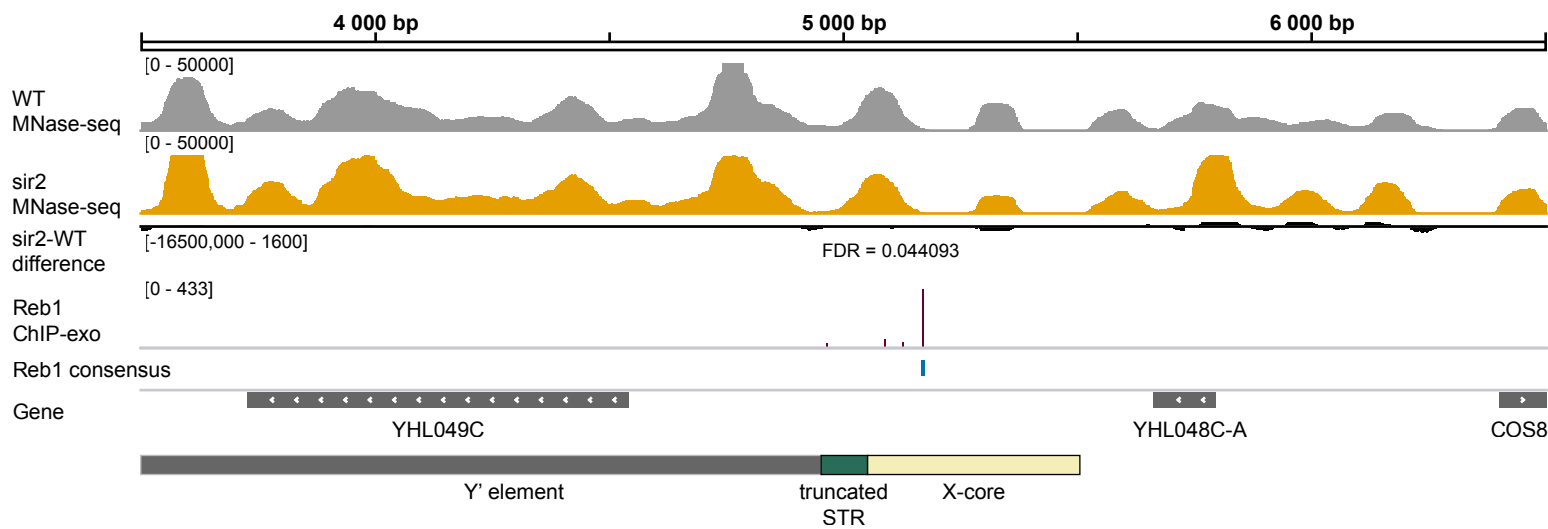

## TEL8R

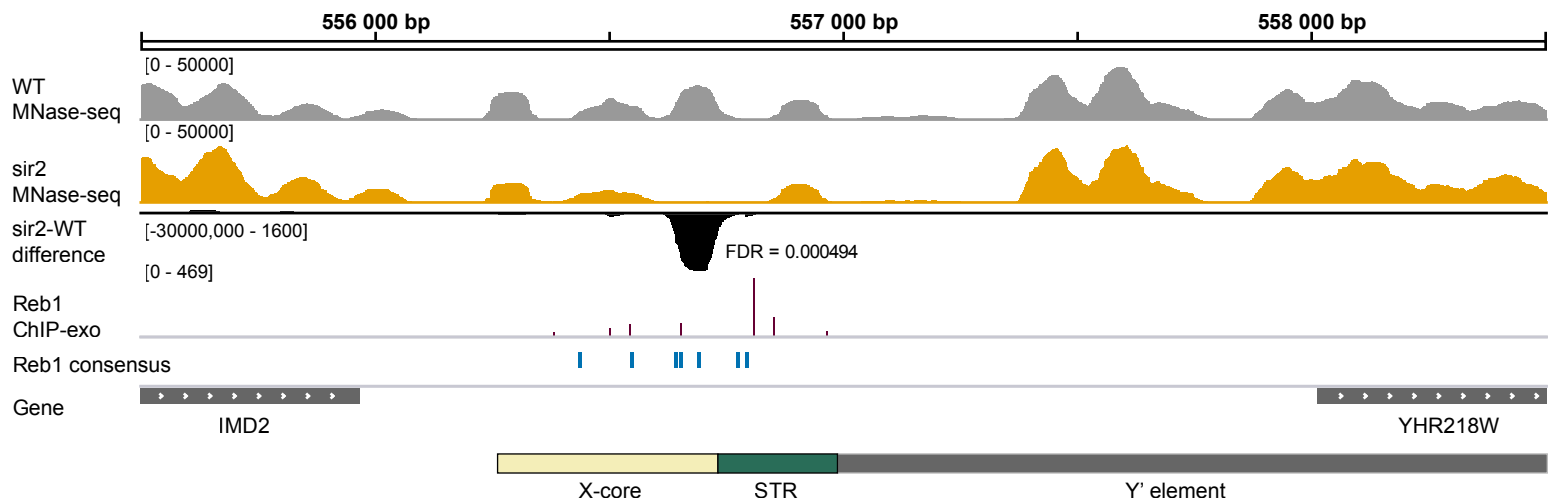

## TEL9L

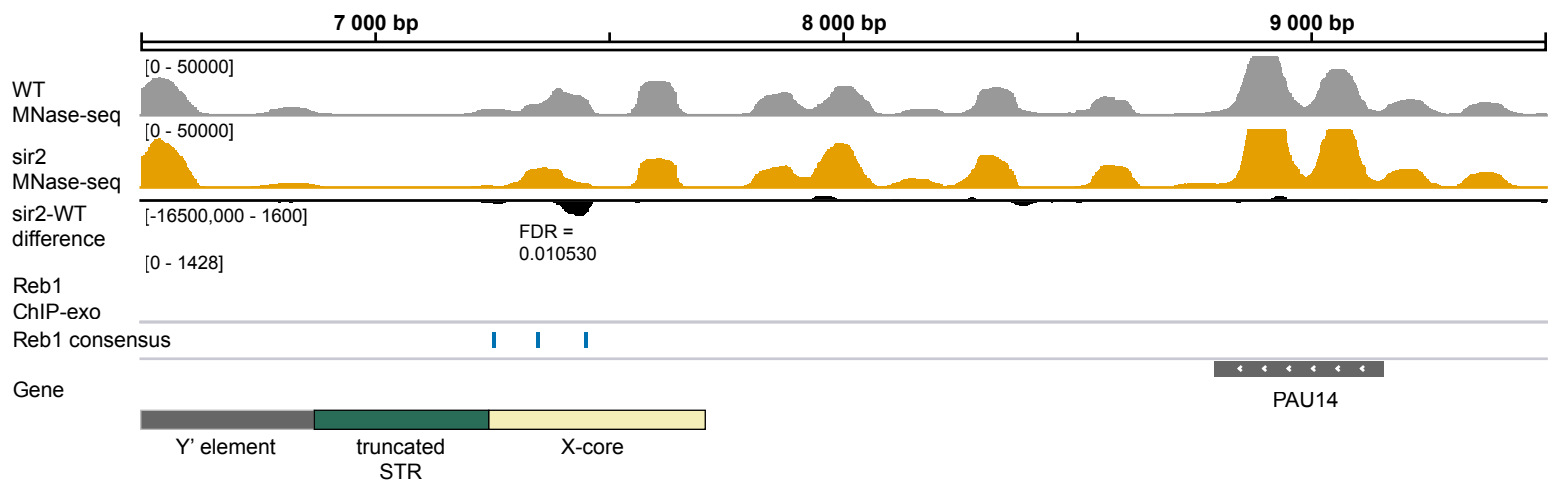

## TEL9R

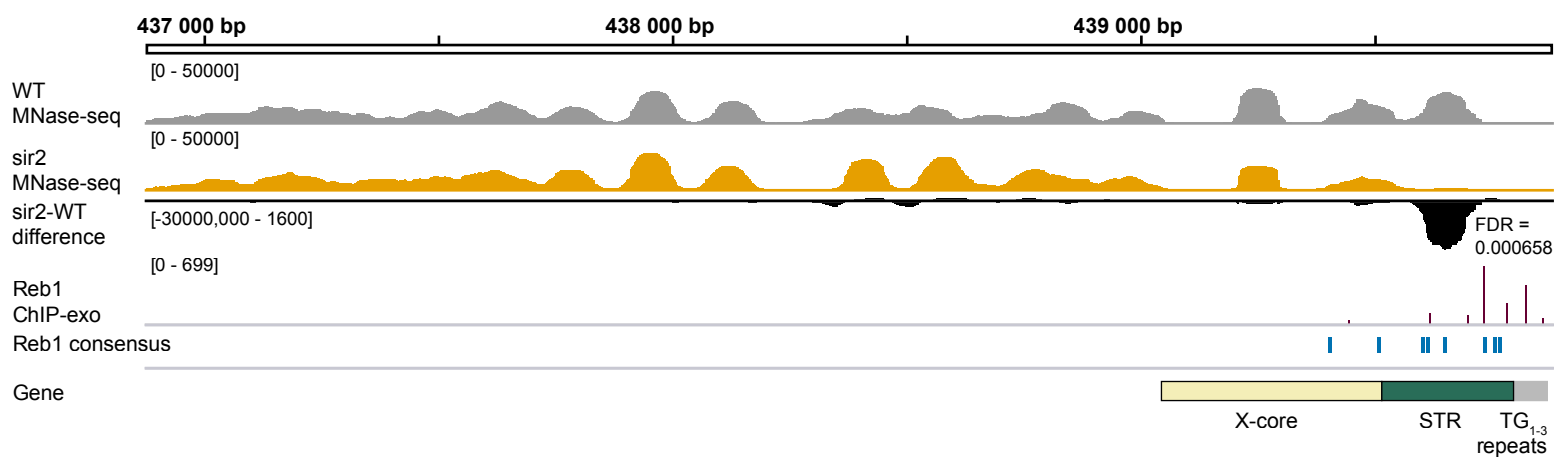

Supplement: S3 Fig — (PDF) [file pgen.1010419.s007.pdf]

## TEL10L

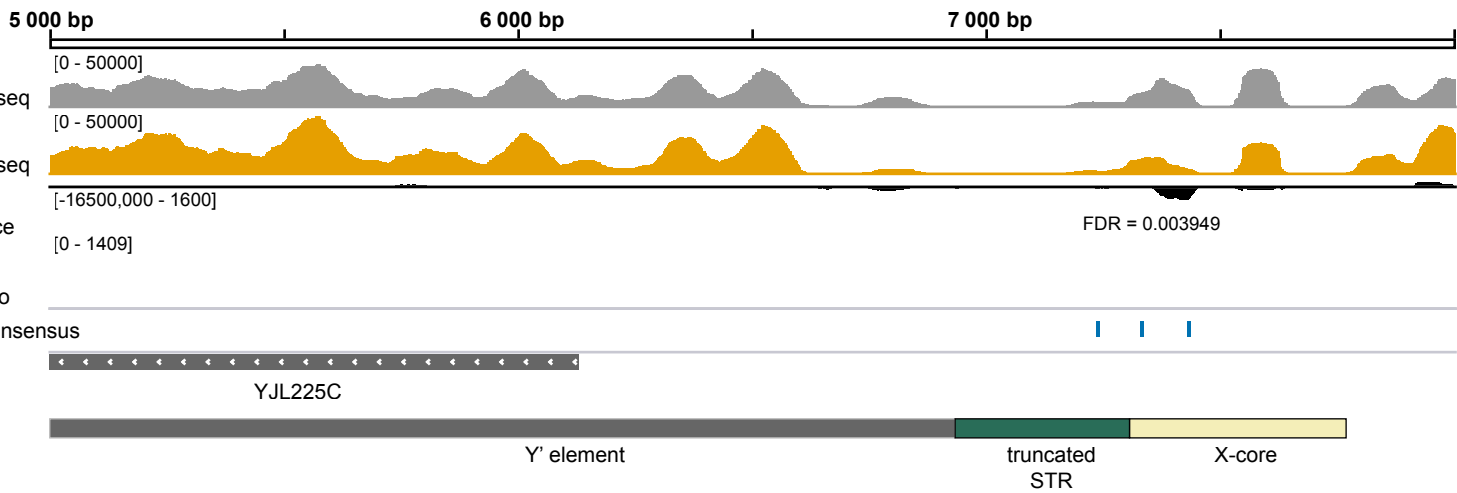

## TEL10R

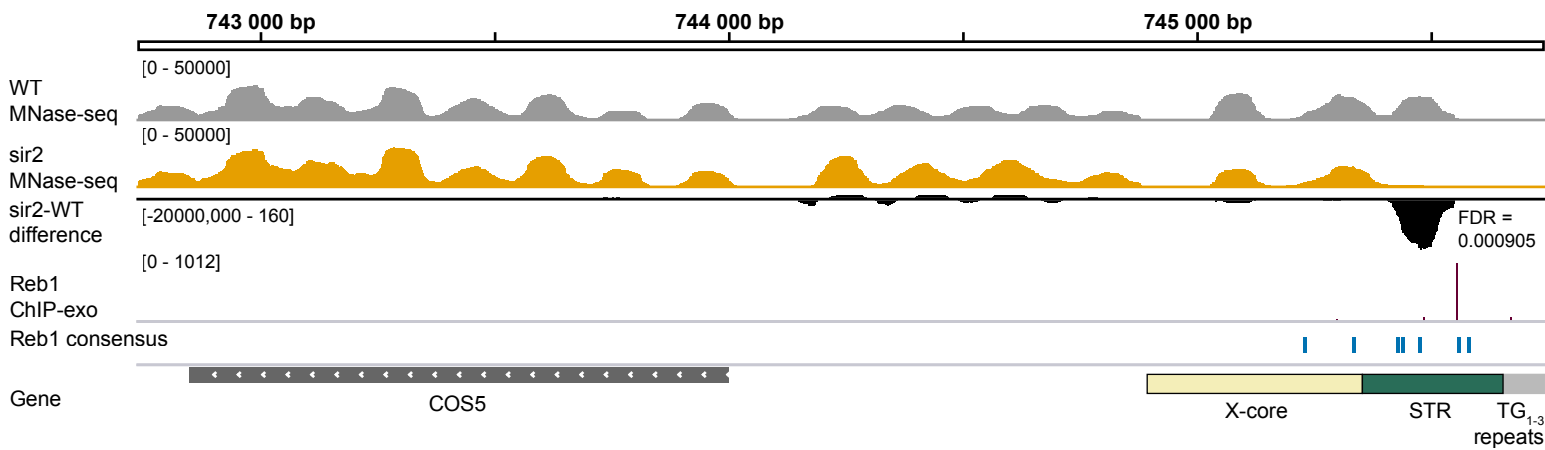

## TEL11L

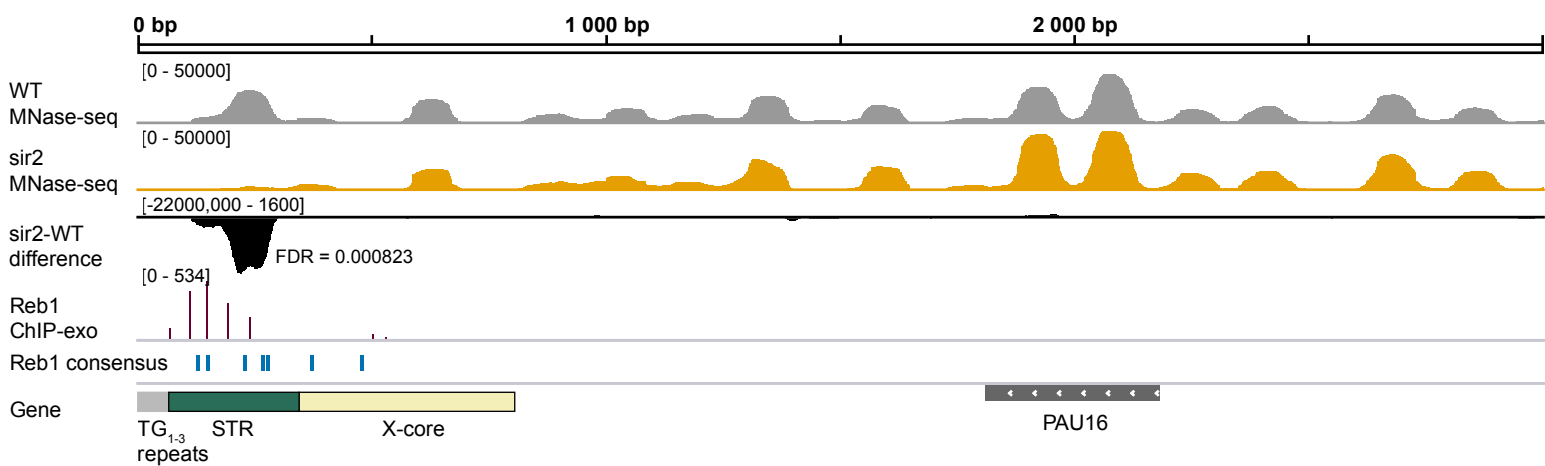

## TEL11R

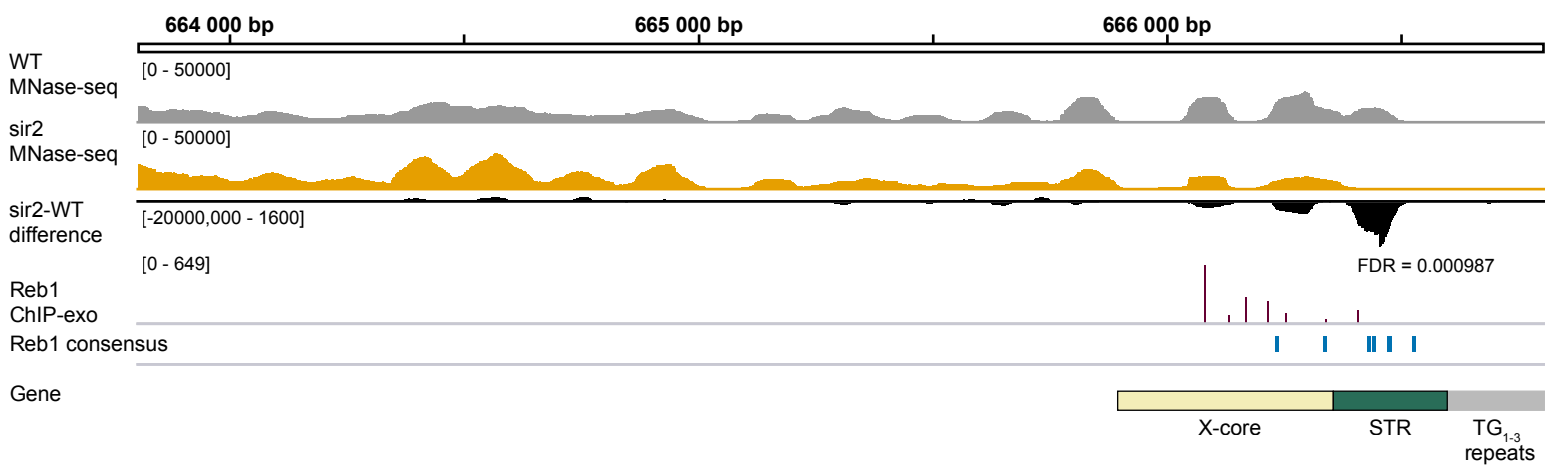

Supplement: S4 Fig — (PDF) [file pgen.1010419.s008.pdf]

## TEL12L

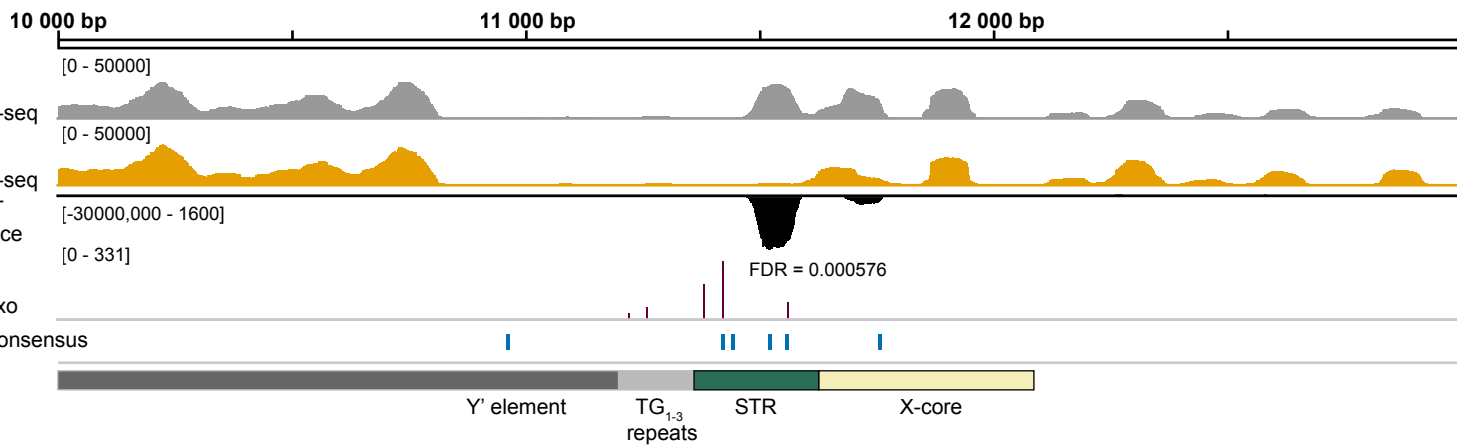

## TEL12R

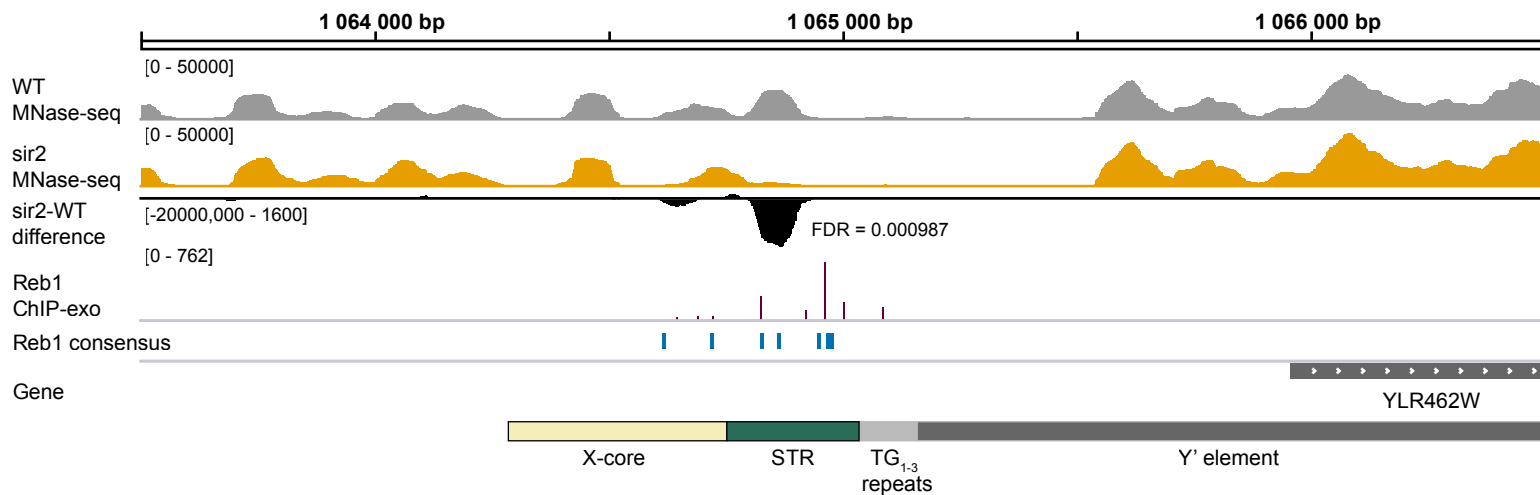

## TEL13L

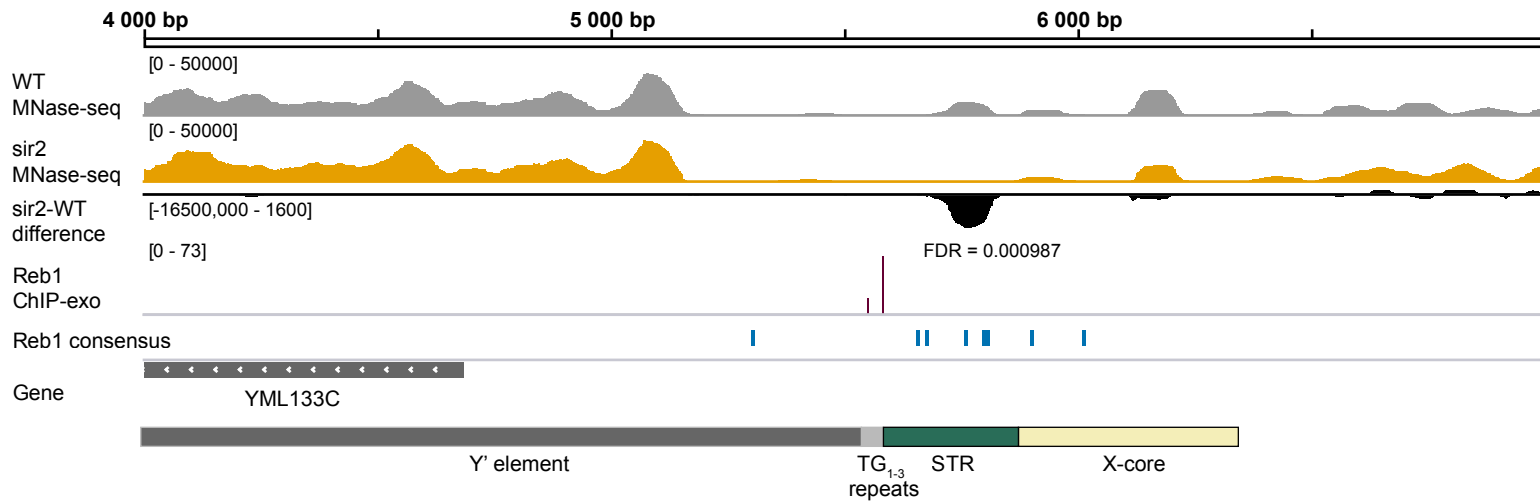

## TEL14L

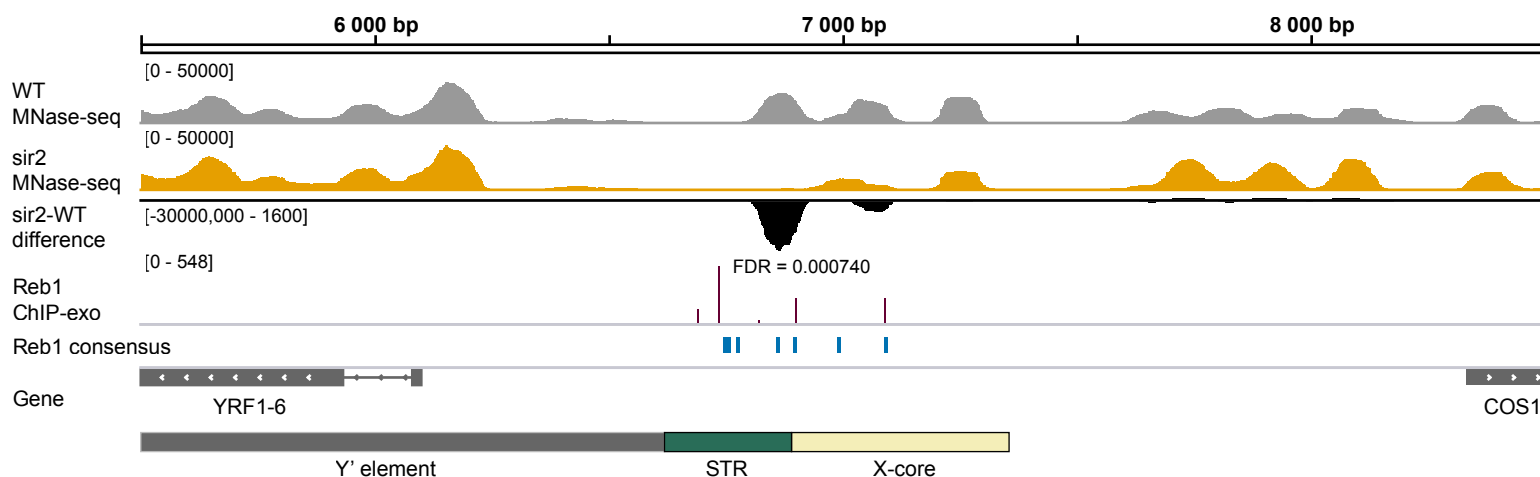

Supplement: S5 Fig — (PDF) [file pgen.1010419.s009.pdf]

# TEL15L

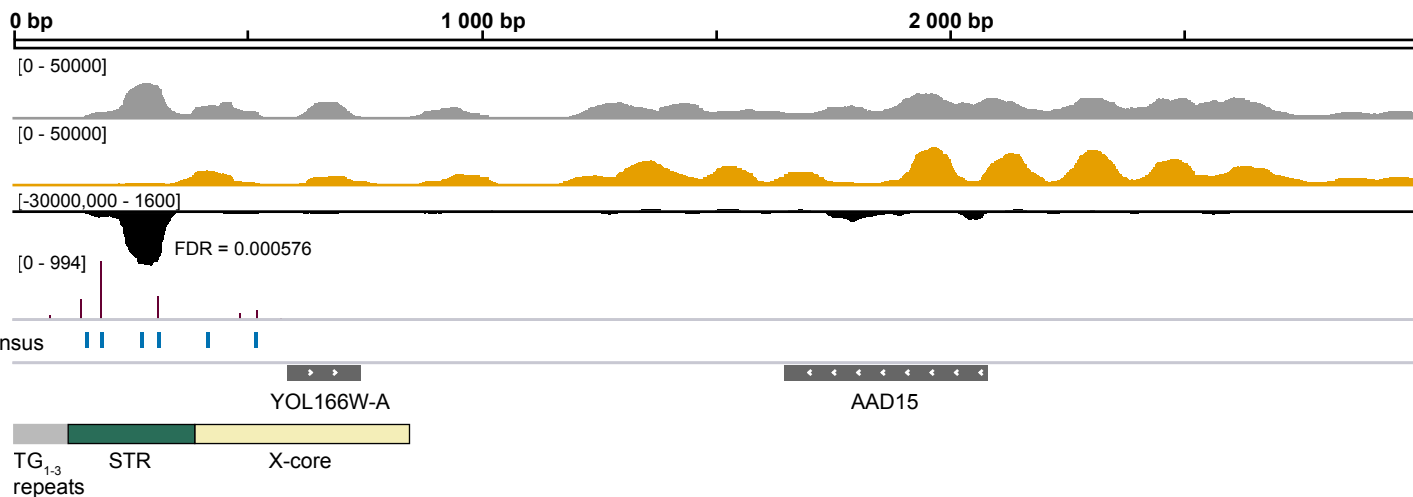

# TEL15R

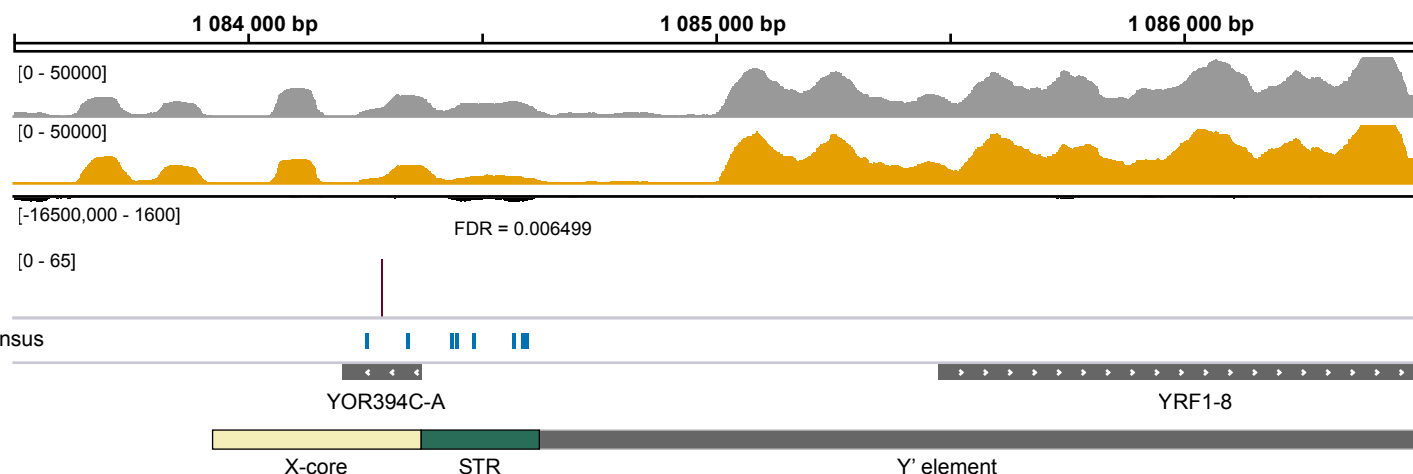

# TEL16L

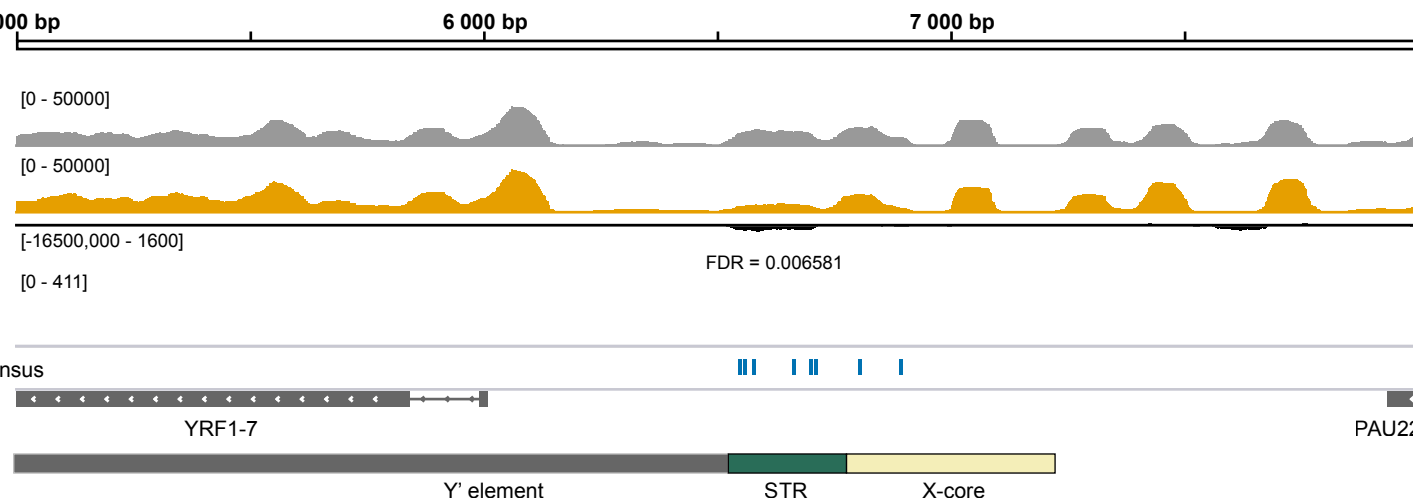

# TEL16R

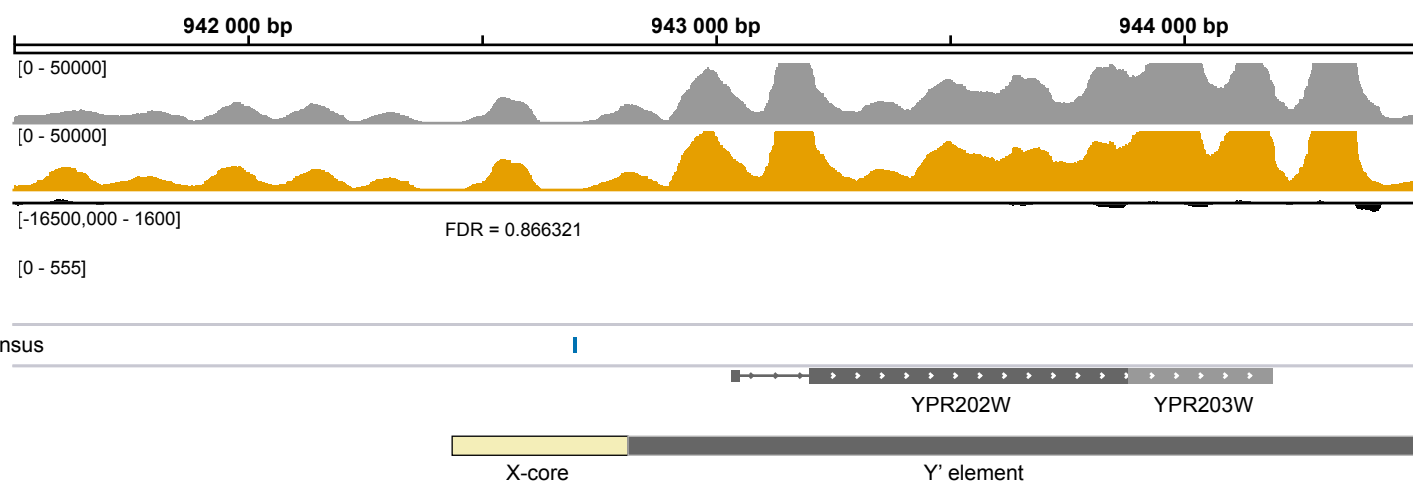

Supplement: S6 Fig — (PDF) [file pgen.1010419.s010.pdf]

## TEL1R

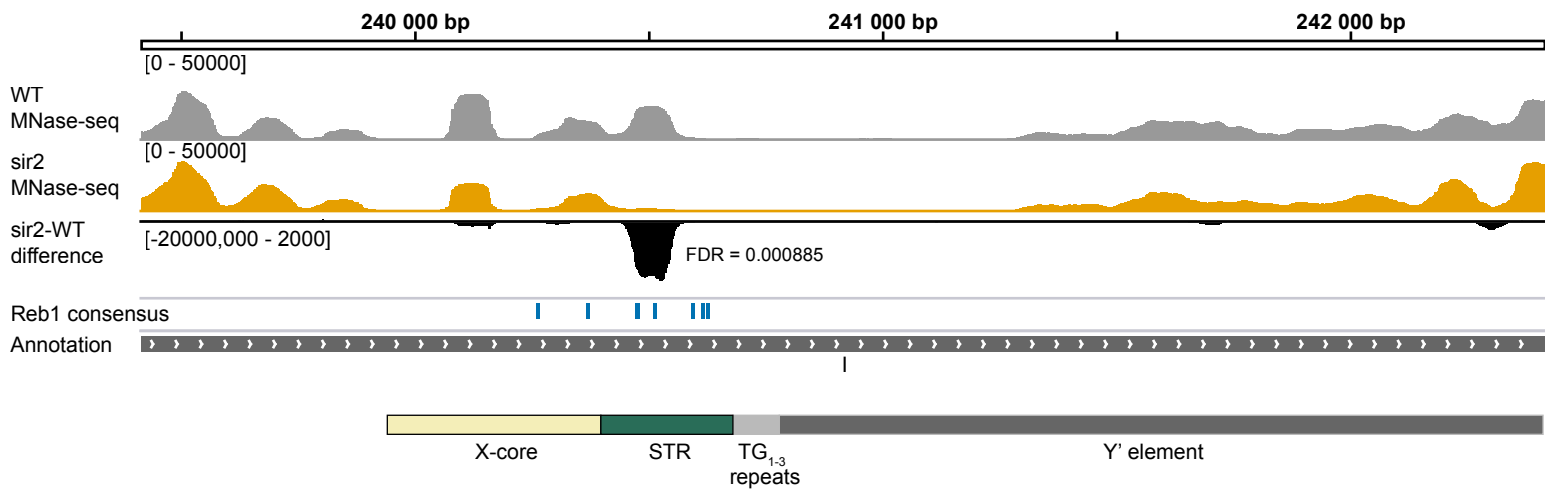

## TEL3R

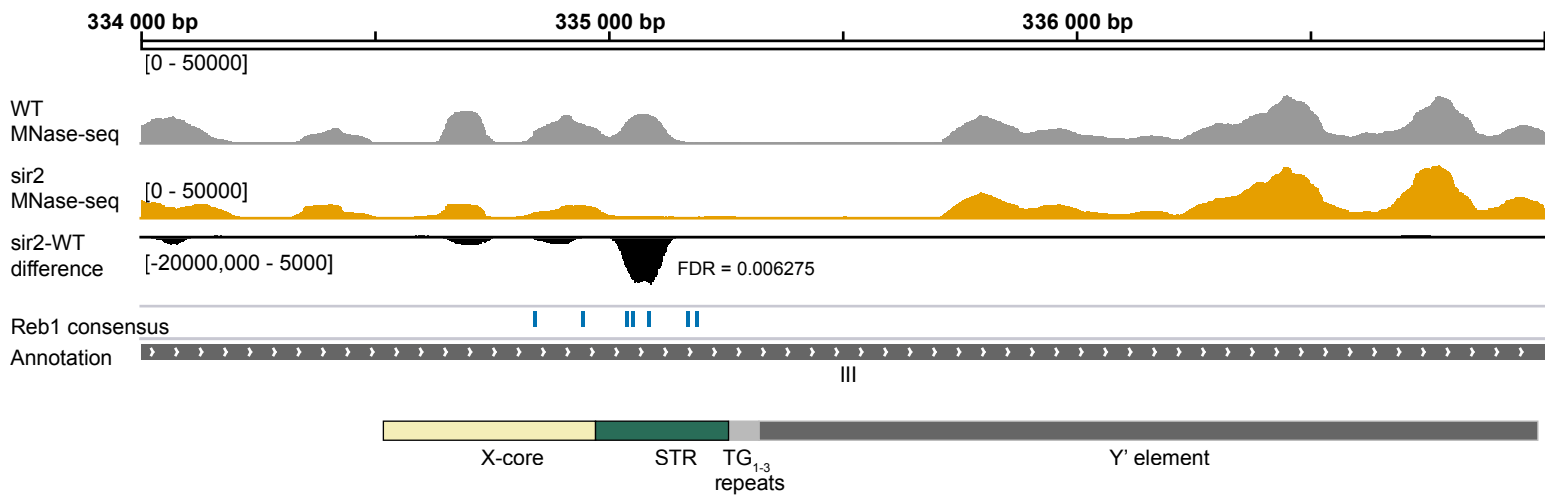

## TEL4L

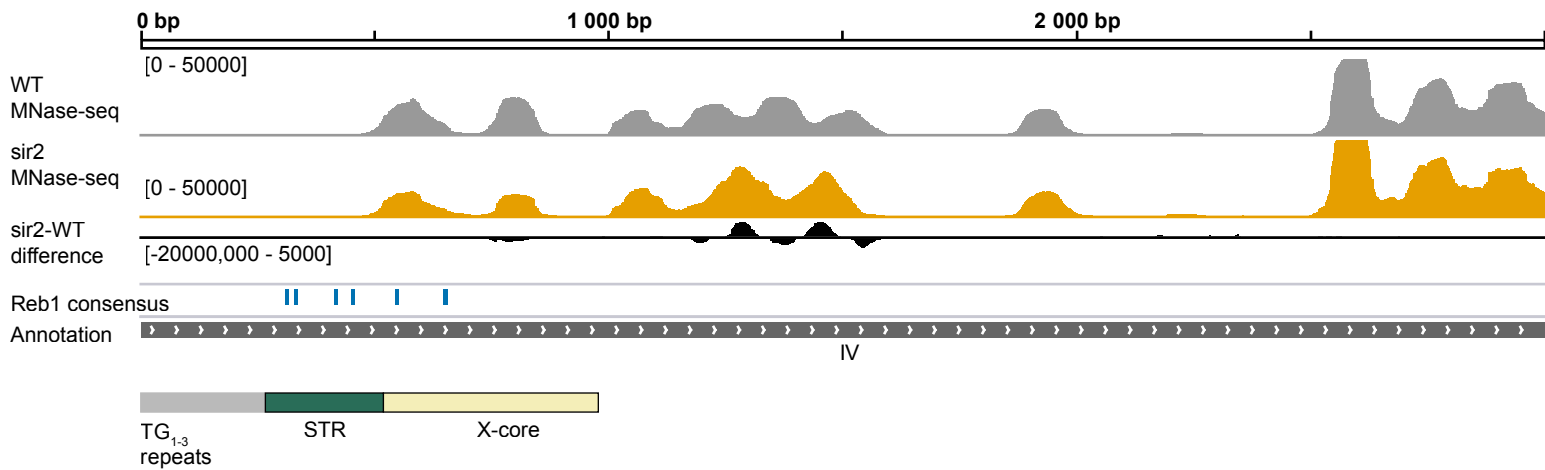

## TEL7L

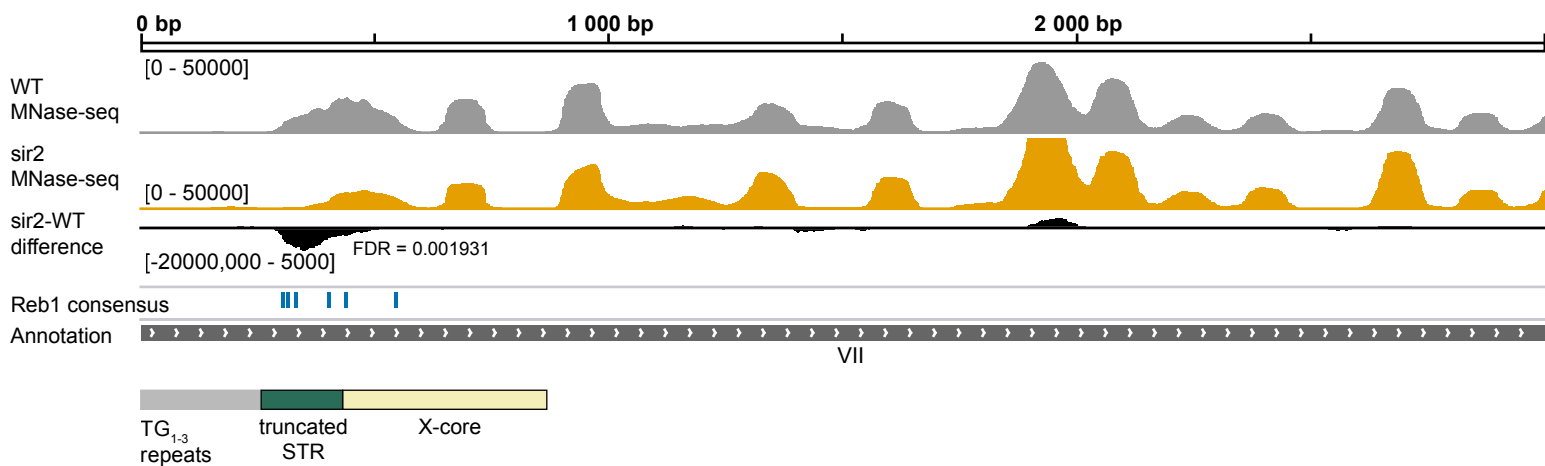

Supplement: S7 Fig — MNase-seq data was aligned to W303 genome. (PDF) [file pgen.1010419.s011.pdf]

TEL13R

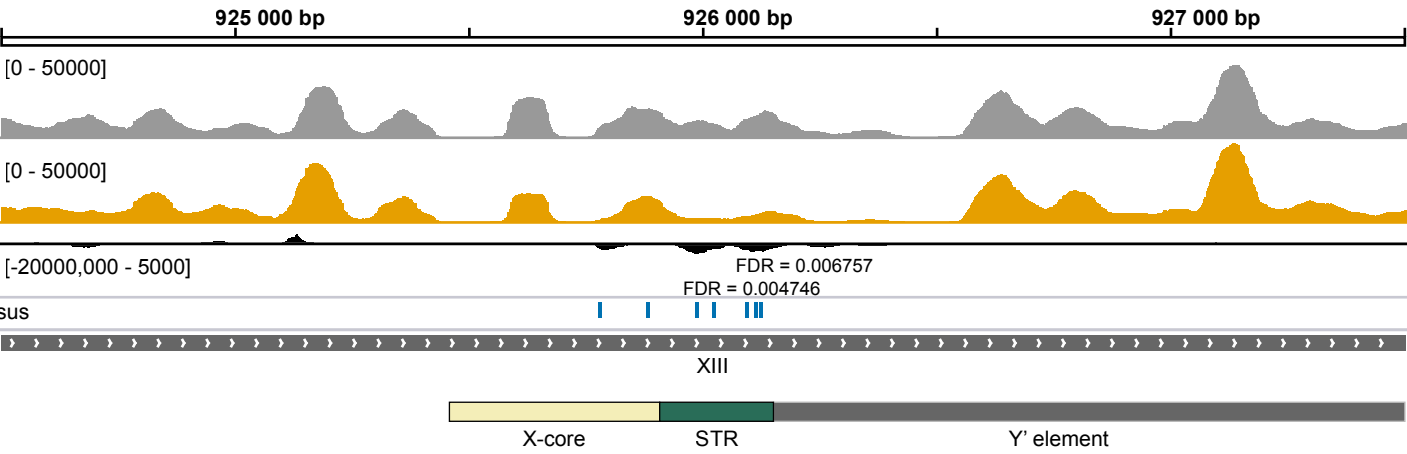

TEL14R

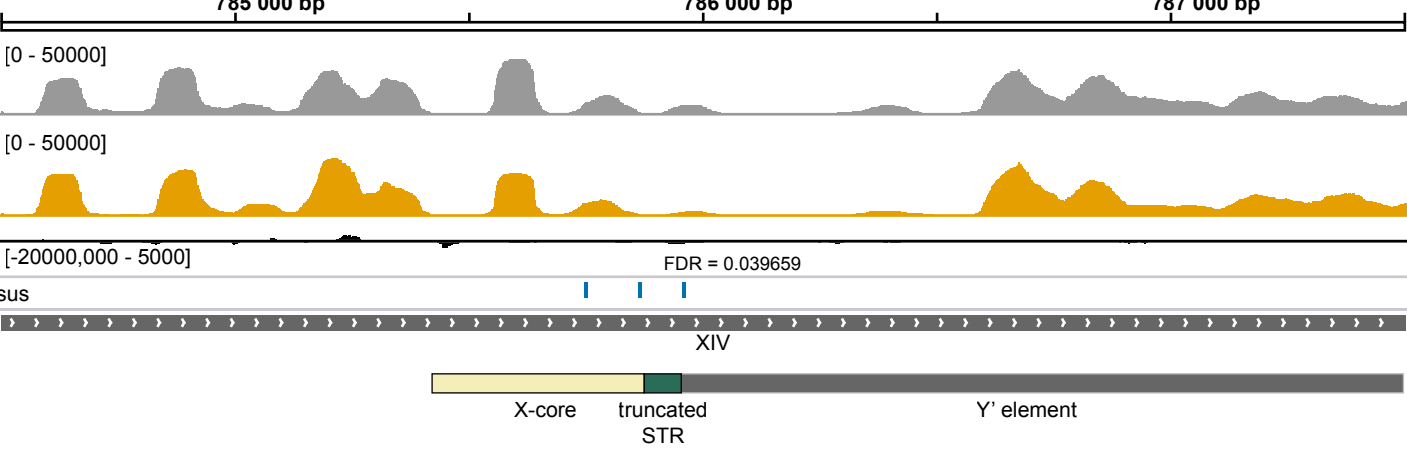

Supplement: S8 Fig — (PDF) [file pgen.1010419.s012.pdf]

**A**      ■ non-*a*/alpha *SIR2*      ■ non-*a*/alpha *sir2*

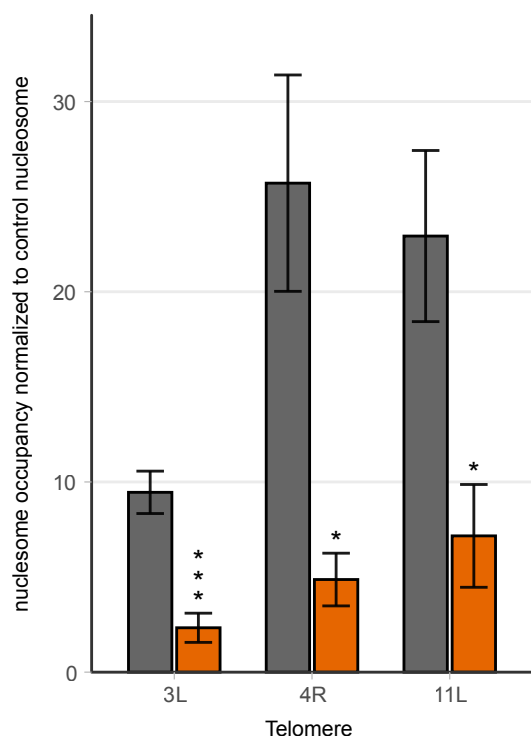

**B**      ■ *WT*      ■ *sir2*      ■ *sir3*

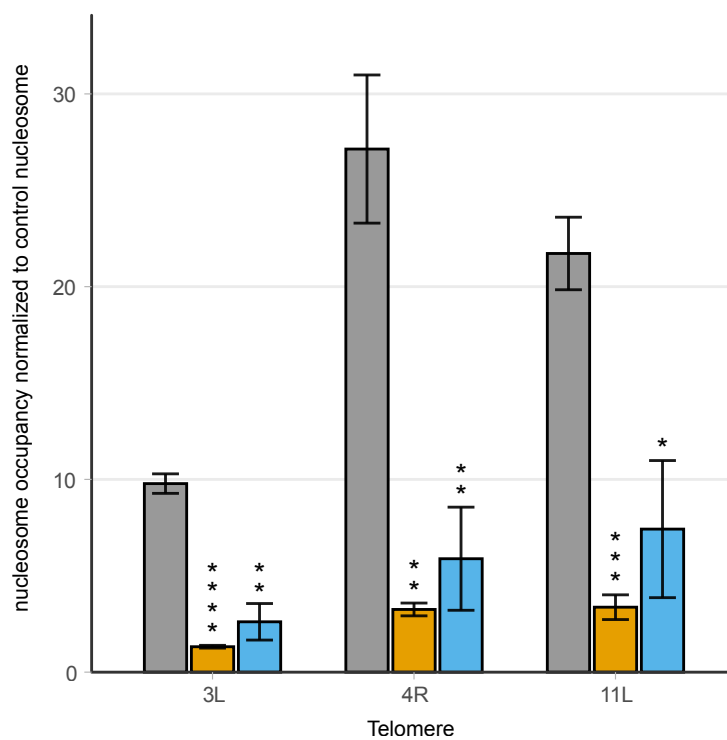

**C**      ■ *H4K16*      ■ *H4K16R*      ■ *H4K16Q*

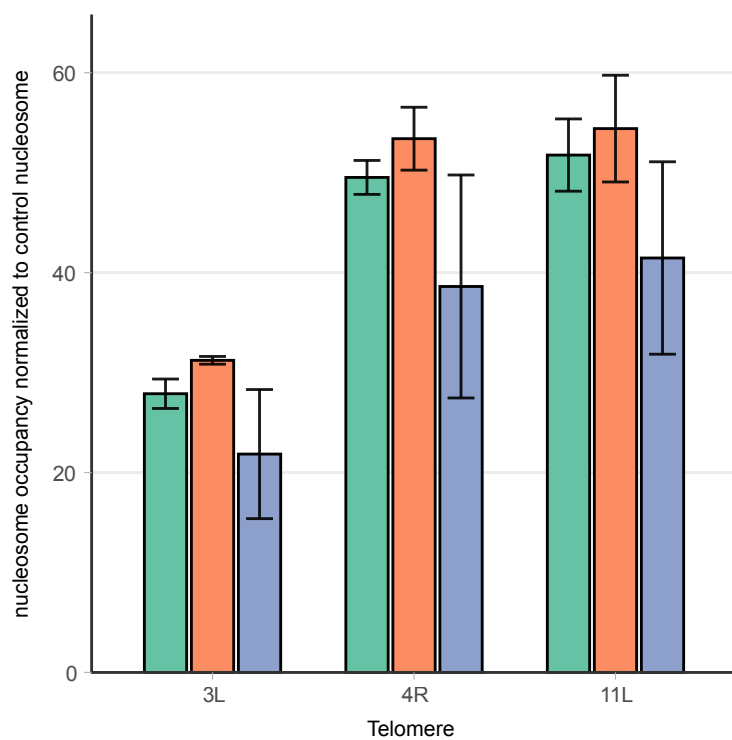

Supplement: S9 Fig — (A) MNase-qPCR of strains deleted for the promoter regions of all 3 mating type loci (mataΔp hmlaΔp hmraΔp) with or without SIR2. (B) MNase-qPCR of WT, sir2 and sir3 strains. (C) MNase-qPCR of strains expressing either WT Histone 4 or mutant alleles mimicking acetylated/deacetylated lysine residue 16. All data are shown as mean ± SEM (n = 3, *p<0.05, **p<0.01, ***p<0.001, ****p<0.0001, ns = not significant). (PDF) [file pgen.1010419.s013.pdf]

**A**

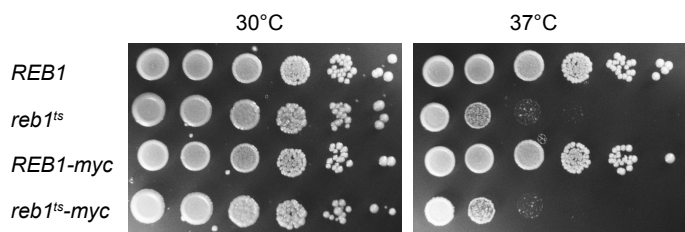

**B**

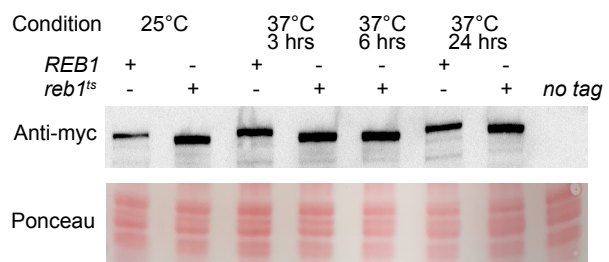

Supplement: S10 Fig — (A) Ten-fold serial dilutions of the indicated strains were spotted on synthetic complete media and incubated at the indicated temperatures for 3 days. (B) Western blot analysis of protein extracts from strains containing the indicated Myc-tagged REB1 alleles at different temperatures and time points using an anti-Myc antiserum (9E11). Lower panel shows Ponceau S staining of the membrane. (PDF) [file pgen.1010419.s014.pdf]

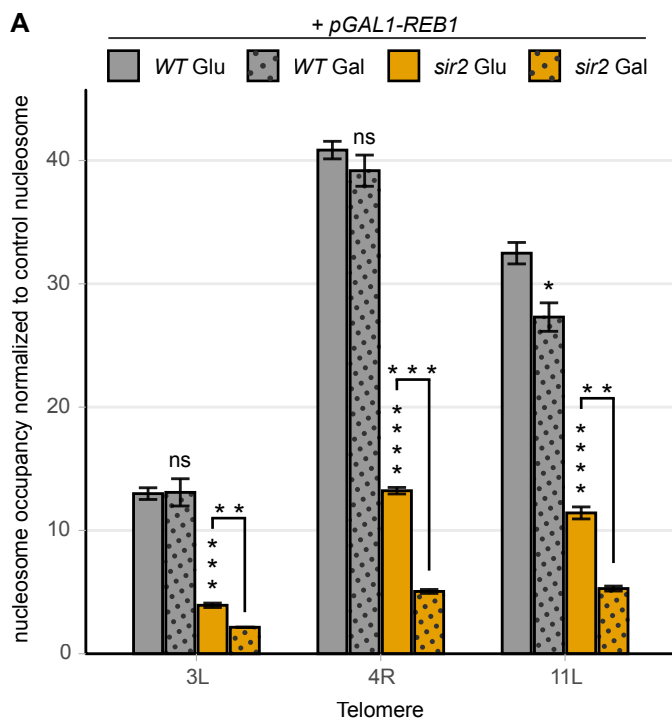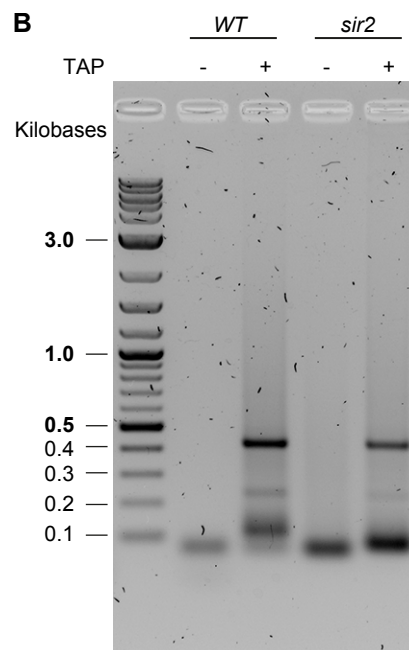

Supplement: S11 Fig — (A) MNase-qPCR of WT and sir2 strains carrying both endogenous REB1 as well as a plasmid-borne copy under the control of the GAL1 promoter. Strains were grown in media containing either 2% glucose (no overexpression) or 2% galactose (overexpression of Reb1, dotted columns). Glu: Glucose, Gal: Galactose (B) Nested PCR of 5′RACE performed either with (+) or without (-) the addition of Tobacco Acid Pyrophosphatase (TAP) to remove the 5′ cap structure of the RNA in the indicated strains to map the TSS of 6R TERRA. (PDF) [file pgen.1010419.s015.pdf]

A

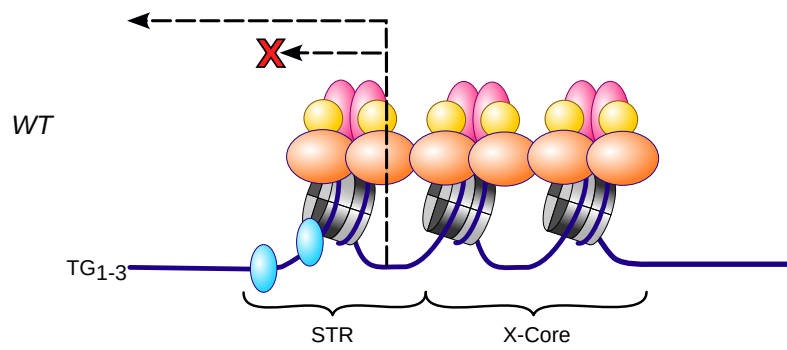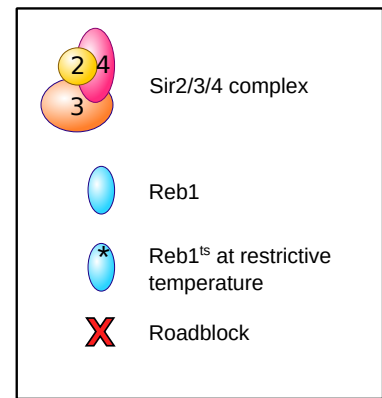

B

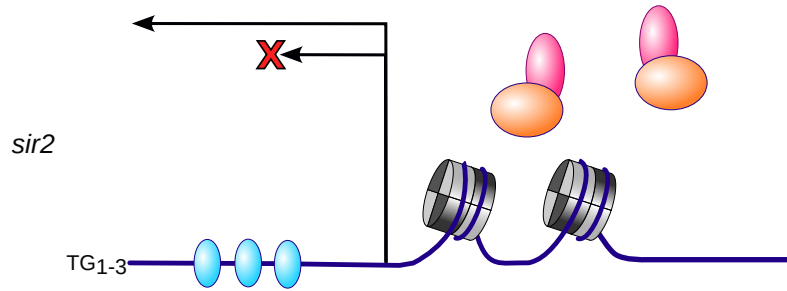

C

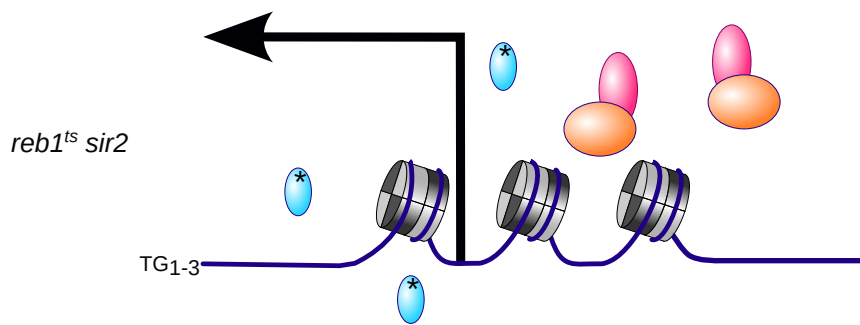

Supplement: S12 Fig — (A) In the WT strain, Reb1 binds to several binding sites in the X-elements, but binds less to the STR-D loci due to competition with the Sir-complex that stabilizes the STR-D nucleosome. This leads to low TERRA steady-state levels as both transcriptional initiation and elongation are repressed. (B) In the sir2 strain, the STR-D nucleosome is lost and Reb1 binding increases. TERRA levels rise due to the increased accessibility for the transcriptional machinery, but Reb1 binding still restricts transcriptional elongation of TERRAs. (C) In the reb1ts sir2 double mutant, the nucleosome is reinstated and TERRA transcription increases synergistically (bottom). See text for further details. (PDF) [file pgen.1010419.s016.pdf]
