# Supplementary material for: Sir2 and Reb1 antagonistically regulate nucleosome occupancy in subtelomeric X-elements and repress TERRAs by distinct mechanisms
Source: PLoS Genet. 2022 Sep 22;18(9):e1010419. doi: 10.1371/journal.pgen.1010419 (PMC9531808; doi:10.1371/journal.pgen.1010419)
Supplement: Attachment — Submitted filename: Response to reviewers.docx [file pgen.1010419.s017.docx]

Thank you for considering our manuscript (PGENETICS-D-22-00717). We note that all three reviewers find the manuscript interesting and worthy of publication pending changes to the manuscript. Reviewer 2 noticed that the MNase-Seq data was not yet available on GEO (GSE195972). We have now made these data publicly available. Below is a detailed list answering the reviewers concerns also indicating changes made to the manuscript. We included three new supplementary figures to address the reviewers concerns (S7, S8 and S12 Fig). In the revised manuscript, changes are labeled in yellow.

Reviewer 1:

A. The authors state “In the W303 strains used by us, TEL1R, 7L, 13R and 14R have deletions in the subtelomeric regions compared to the reference strain (S288C), partly explaining the lack of read”.

   This got me a bit worried. How can the authors be sure that other conclusions are not impacted by mapping onto S288C genome?
   There is a full reference w303 genome:  Whole-Genome Sequence and Variant Analysis of W303, a Widely-Used Strain of Saccharomyces cerevisiae. Matheson K, Parsons L, Gammie A. G3 (Bethesda). 2017 Jul 5;7(7):2219-2226. doi: 10.1534/g3.117.040022.
    
   Which makes it a bit curious that the authors did their alignments to the S288C genome, might be a good idea for them to double check that their conclusions hold with the W303 reference genome.
   There are issues with aligning our MNase-Seq reads to the W303 genome assembly even if the S288C and W303 genomes are >99.9% identical. The release 64 assembly (S288C) used as a standard option by most researchers has a superior annotation compared to the semi-automated annotated W303 assembly. A further complication is that the ChIP-exo data set for Reb1 is based on release 64, and Reb1 occupancy is presented as exact genome coordinates. Since the genome coordinates change between strains, we cannot use the ChIP-Exo data tracks and generate the meta-X-element analysis, including the heat map of Reb1 binding, if the MNaseSeq reads are aligned to the W303 genome. In addition, we use the SGD coordinates (based on release 64) to localize where the X-core and STR-elements begin and end.
   We nevertheless aligned the reads to the W303 genome and analyzed the resulting data to explore if we could obtain nucleosome tracks for the six telomeres that contained few or no reads when aligning to R64-1-1 (TEL1R, 3R, 4L, 7L, 13R and 14R). We obtained nucleosome tracks for all six telomeres and manually determined the locations of the X-core and STR-elements. For 1R, 3R, 7L and 13R we found significant nucleosome differences in the STR-elements, but not for 4L and 14R. For 14R this was likely due to a truncated STR-element and 4L did not have a nucleosome covering the STR-element even in the *WT*. Hence, the alignment using the W303 assembly reinforced the conclusions of the manuscript. We included these nucleosome tracks as S7-8 Fig and changed the text accordingly (line 177-). Given the complications listed above, we did not use the W303 genome for analyzing the other telomeres. The W303 alignments were submitted to GEO.
B. The model proposed by the authors suggests a competition between Reb1 and Sir2. The authors show increased Reb1 binding in Sir2 mutants, but they should also test the opposite: ie, is there increased Sir2 binding in the reb1-ts strain. Alternatively, the authors should clearly define this as a remaining caveat/future direction and modify the relevant text.
   Sir2 does not bind DNA directly, but associates with nucleosomes as part of a Sir2/3/4-complex. STR-D nucleosome occupancy increased less than two-fold in the *reb1^ts^* strain compared to the *REB1* strain at the restrictive temperature (Figure 3B). Previous ChIP-Seq experiments of Sir-proteins has demonstrated broad binding peaks with erroneous binding to so-called “hyper ChIPable loci” (Teytelman, 2013, PNAS, 46, 18602-). This made us hesitate to investigate increased Sir2 binding to X-elements in the *reb1^ts^* strain. However, we made an addition to the discussion indicating that this would be a sensible experiment (line 469-).
C. Line 266: “We next set out to test the effect of depleting Reb1 on nucleosome occupancy. To this end, a temperature-sensitive allele of Reb1 was generated (see materials and methods).”
   Suggest change to:
   “We next set out to test the effect of depleting Reb1 on nucleosome occupancy. Since Reb1 is essential, a temperature-sensitive allele of Reb1 was generated (see materials and methods).”
   We changed the text as suggested by the reviewer (line 283-).
D. The authors could include an overall model for the Reb1 and Sir2 function in nucleosome positioning and TERRA transcription.
   We included a model in the S12 figure.
E. I recommend switching the order of Figure 5A and Figure 5B.
   We switched the order of figure 5A and 5B.
F. In the discussion the authors mention that Sir2 could be targeting other lysine residues, but could clearly mention the possibility that Sir2 is working via targeting of both histone and non-histone proteins.
   We added a short comment on this in the discussion (line 489).

Reviewer 2:

1. I appreciate that the authors addressed the issue of the derepression of the mating-type loci HML/HMR in the sir2∆ mutant in Fig. S7A.
   Thank you, other phenotypes of *sir* mutants (sterility, NHEJ deficiency) are due to indirect regulatory effects so we were motivated to perform the experiment.
2. Given that Y’ elements share a high percentage of sequence identity, how were the reads accurately mapped to specific Y’ elements?
   Most reads from Y’-elements had multiple best-score alignments. Reads with multiple best-score alignments (multi-mapping reads) were reported as one randomly selected alignment to allow for analysis of repetitive regions while reducing the overrepresentation of individual homologous regions in read pileups.
3. Is there an accurate W303 genome that the authors could have used to map the reads, instead of R64-1-1? They could have had a better view of subtelomeres 1R, 7L, 13R and 14R See answer A to reviewer 1.
4. The sir2∆ results in Fig. 2B do not seem to be referenced in the text.
   We agree and modified the text: “Quantitative PCR using primers specific to the STR-D loci at TEL3L, 4R and 11L showed that the DNA resistant to MNase digestion was efficiently immunoprecipitated by the histone H3 antibody (Fig 2B) from the *WT*, but not from the *sir2* strain. Hence, the MNase-resistant DNA at the STR-D loci were nucleosomes.”(line 177-)
5. Lines 228-230 and 463-465: based on the result using the mutant sir2-N345A, the authors suggest that the catalytic activity of Sir2 might be important for nucleosome stabilization. However, Armstrong et al. (ref 34) showed that this mutant fails to properly localize to the telomere. The reduction of nucleosome occupancy in this mutant might not be directly due to a defect in the catalytic activity, but rather due to a decreased recruitment of Sir2.
   We have modified the discussion on the *sir2N345* mutant, attempting to clarify the issue (line 481-). Since Sir2-mediated deacetylation of histones (mostly H4K16) is important for the spreading of silent chromatin it is expected that less Sir2N345A (and Sir3/4) would be recruited to silent DNA, but not necessarily to the nucleation point, i.e. the Orc and Abf1 binding sites in the X-core. Thurtle and Rine (ref 25) showed substantial recruitment of Sir3/4 to the silencers in a *sir2N345* mutant background, but much less spreading to internal positions of *HML* and *HMR*. Armstrong et al. actually only showed decreased Sir3 recruitment to Tel6R in the *sir2N345* background, but did not show the data for Sir2 (ref 37).
6. Lines 144-146: although it is fair that the authors wish to present their results for nucleosome regulation at other loci in another publication, it would be nice to know if the mechanism shown here is specific to subtelomeres or more general. I do not know whether referring to unpublished data (which is no longer a common practice) or showing a figure without revealing too much would be a solution.
   We considered including nucleosome changes in the regulatory regions of cell-type specific genes, but decided that this would distort the focus of the manuscript. Importantly, these changes are indirect, the result of the a1/alpha2 and alpha2/Mcm1 transcriptional repressors, presumably with the help of the chromatin remodeler Isw2. We observe significant nucleosome changes (both positioning and occupancy) at 8 a-specific genes and 15 haploid-specific genes. The nucleosome changes are centered on the binding sites for the transcriptional repressors and for some loci, the recombination enhancer and HO-promoter for example, the changes are quite complex. These data require further analysis and deserve to be presented by themselves. An igv snap-shot of the *STE4* locus, an a1/alpha2-repressed gene encoding the beta-subunit of the heterotrimeric G-protein, required for mating pheromone signaling, is given as an example below. Note the red bar, which is the a1/alpha2 binding site and the increased nucleosome occupancy induced by repressor binding. The S1 Table lists the most significant nucleosome changes in the regulatory regions of cell-type specific genes and the full data set is freely available in GEO.

7. Fig. S8B: can the authors comment on the slight change in mobility between Reb1 and Reb1ts?
   The *REB1* and *reb1^ts^* alleles were epitope-tagged individually, confirmed by PCR and Western-blotting. We hypothesize that the slight mobility difference is due to different numbers of Myc-tags fused to *REB1* and *reb1^ts^*, respectively. The template used has 13 Myc-tags, but internal recombination events in this repetitive stretch of DNA can result in deletions.
8. Fig. 4C: the transcription termination assay demonstrates that the X-element can terminate transcription. However, whether this property is Reb1-dependent was not really assessed in a convincing way. The authors used the reb1ts mutant at permissive temperature, arguing that it has a partial loss of function, and showed that it is less efficient than wild-type to terminate transcription. Ideally, the authors could perform the assay with reb1ts at a semi-restrictive temperature (or over a range of temperatures), so that cells would still grow rather well without Cu2+ but would show a stronger growth defect in the presence of Cu2+. Alternatively, the assay could be performed with a X-element mutated for its Reb1 binding site(s).
   We did perform the termination-assay at several temperatures, but chose to present the data from 30 degrees as higher temperatures (already at 32 degrees) started to affect the growth of the *reb1^ts^* strain, making interpretation of the data difficult. Others (ref.42) demonstrated that a single Reb1 binding site was sufficient to terminate transcription and that mutating this site, abolished termination. The 3L X-element contains five consensus binding sites for Reb1 and several close matches, so it is not trivial to change all of these sites.
9. Lines 289-290: Dr Krallis (from the Wellinger lab) did experiments testing Reb1’s role in regulating TERRA. They are reported in her PhD thesis: <https://savoirs.usherbrooke.ca/bitstream/handle/11143/17175/Krallis_Alexandra_MSc_2020.pdf?sequence=1&isAllowed=y>
   But their work has not been published in a peer-reviewed journal yet, to my knowledge.
   Yes, we are aware of this M.Sc. thesis, but as the reviewer points out, this work has not been peer-reviewed.
10. It is not straightforward to conclude about the lack of correlation between telomere length and TERRA expression using mutants as examples, due to potential indirect effects of the mutants.
    We agree and avoid over-interpreting the telomere length data. Our only conclusion is that a fully functional Reb1 is required for maintaining normal telomere length.
11. In most graphs, statistical tests are shown for comparison against the wild-type condition, but sometimes additional tests can be useful, for example in Fig. 5A “amplicon 2” between URA3::TEL3L-X and URA3::TEL3L-X sir2∆. By the way, can the authors explain this observation of higher nucleosome occupancy in the absence of Sir2 at the URA3 locus?
    We added more statistical comparisons in figure 5 and we do not know why there is higher nucleosome occupancy in *sir2* at the *URA3* locus.
12. A prediction regarding nucleosome occupancy affecting TERRA expression is that at an X-element where sir2∆ does not show nucleosome depletion, e.g. 5R, TERRA should not be increased upon SIR2 deletion or Reb1 delocalization (using reb1ts at restrictive temperature). Did the authors test this prediction?
    The first strand cDNA synthesis for measuring TERRAs uses a primer complementary to the terminal repeats (since TERRAs are defined as transcripts including terminal repeats) and the qPCR primers for detecting TERRAs from individual telomeres reside in the X-elements. Since the Y’-elements always are telomere proximal compared to the X-elements, the first strand synthesis must extend >6kb in order to detect TERRAs from Y’-telomeres. This technical limitation makes it difficult to reliably detect TERRAs from Y’-containing telomeres. The telomere suggested, 5R, has a Y’ and has a significant lower nucleosome occupancy in *sir2* (FDR=0.0018). We have not tested the suggested prediction because we did not observe any X-only telomeres that does not have a Sir2-stabilized nucleosome.
13. Line 25: “Reb” should be “Reb1”.
    Thank you for noticing, our mistake.
14. Lines 95-96: for the discovery of TERRA, Azzalin et al. 2007 Science should be cited in my opinion, even though the work was done in mammalian cells.
    We agree and included the suggested reference.
15. Fig. 1C: maybe the data points corresponding to the same chromosome extremity in WT vs sir2∆ could visually linked with a straight line for example? The points corresponding to X-core of to STR-D could also be distinguished using different colors.
    We changed symbols in figure 1C, using rectangles for the X-core reads and circles for the STR-reads. We did not draw a line between the values belonging to the same telomere, as this made the figure too busy. However, the MNaseSeq reads are in the S1 Table and the FDR values in S1-6 Fig.

Reviewer 3:

1. TERRA R-loops have been shown to delay the onset of replicative senescence. Can the authors test whether the reb1-ts strain delays replicative senescence, as there should be more TERRA R-loops present due to the lac of termination. Testing Reb1 overexpression as well would be interesting in this context.
   From a strict telomere biology point of view, it is reasonable to assume that replicative senescence would be delayed in the *reb1^ts^* strain because of increased TERRA levels/R-loops. However, Reb1 affects many genes, i.e. it is necessary for proper snoRNA expression, for correct nucleosome positioning and expression of RSC-regulated genes, for terminating cryptic transcripts and possibly Pol1 transcription, etc. Hence, the *reb1^ts^* strain presumably displays numerous upsets in gene expression unrelated to TERRAs. Due to these pleiotropic effects, the *reb1^ts^* strain probably has a shortened replicative lifespan (RLS). As for overexpression of Reb1, similar problems with interpretation of effects on RLS arise. It would be very difficult to distinguish a shortened RLS due to decreased TERRAs from effects due to increased occupancy of the numerous functional Reb1 binding sites elsewhere in the genome.
2. TERRA is regulated in a cell cycle dependent manner in both yeast and human cells as demonstrated by the Lingner and Luke labs. Does this correspond at all with Reb1 occupancy? i.e. is Reb1 at telomeres in G1, but absent in early S phase when TERRA gets transcribed (have a look at Graf et al, alpha factor and HU experiments)
   Looking at the data in Graf et. al., 2017 (Figure 1), TERRA levels increase 2-4 fold in late S-phase compared to G1. Inactivation of Reb1 resulted in much larger increases in TERRA levels (> 100-fold, Table 1). Therefore, if decreased Reb1 occupancy in S-phase would explain the cell cycle differences in TERRA levels, it would most likely be a rather subtle effect. As we argue in the manuscript, due to the clusters of occupied Reb1 binding sites in X-elements and the limitations of the ChIP-technique, it is difficult to detect subtle differences in Reb1 occupancy at these loci. According to cyclebase.org, the peak expression of the *REB1* mRNA is in late S/G2, which seems to be the opposite of what would be expected for such a model.
3. I am a bit worried about the temperature effects on TERRA levels as temperature drastically affects TERRA levels. Can you also see the nucleosome effects/TERRA effects with a Reb1 degron, hence avoiding temp shifts.
   We tried an auxin-inducible degron, but this did not work in our hands, i.e. the strains remained viable at high concentrations of auxin and we did not detect decreased levels of Reb1-AID. The control (*REB1*) and *reb1^ts^* strains were investigated at both the permissive and restrictive temperatures. Since the only difference between these strains was the *REB1* allele it was possible to conclude that the differences observed in TERRA levels were due to loss of Reb1 rather than temperature itself. We argue that temperature sensitive alleles are less prone to off-target effects compared to other inducible depletions since no chemicals have to be added to the cells
4. Finally, is the increase in telomere length due to telomerase or HR?
   We have not investigated if the increased telomere length observed in the *reb1^ts^* strains is due to recombination or increased telomerase activity. We plan to study *reb1^ts^* *tlc1* double mutants exploring if either the frequency and fraction of type 1 and type 2 survivors are affected. These experiments are labor intensive and we plan to present them in a later study. As pointed out by reviewer 2, it is problematic to correlate TERRA levels with telomere length using mutations in genes that induce pleiotropic effects.
